# The ‘vulnerability paradox’: how institutional legacies shaped Colombia's response to Venezuelan displacement

**DOI:** 10.1111/disa.70047

**Published:** 2026-02-13

**Authors:** Nieves Fernández‐Rodríguez

**Affiliations:** ^1^ WZB – Berlin Social Science Center Germany; ^2^ Nebrija University Spain

**Keywords:** crisis, forced displacement, institutional legacies, vulnerability paradox

## Abstract

Colombia's response to Venezuelan displacement—driven by economic collapse, political instability, and humanitarian need—through the Temporary Protection Status programme has been praised internationally for its inclusive approach and positive effects on both migrants and host countries. This research argues that the adoption of a crisis lens is the key to understanding how such an effective response was possible. Based on 40 semi‐structured interviews with policymakers and experts, as well as a review of literature on internal forced displacement policy, this article highlights how Colombia leveraged institutional legacies from its internal conflict and displacement crises to shape its response. The findings identify five capacities that were transferred across crises: management of international aid; coordination among institutions; population registration; integration of external models; and understanding of the nature of the border. The article contributes to the literature on large‐scale displacement by emphasising the importance of institutional memory in shaping responses and suggests the presence of a ‘vulnerability paradox’: how countries with histories of adversity can build resilience and crisis management capacities.

## 1 INTRODUCTION

Since 2015, the displacement of more than 7.9 million Venezuelans has evolved into one of Latin America's most pressing humanitarian crises (UNHCR, 2024). This exodus was fuelled by economic collapse, political turmoil, and acute shortages of basic goods—conditions that stemmed from the sharp decline in oil revenues following the end of the commodities boom, unsustainable debt, fiscal mismanagement, and the increasing militarisation of governance of marginalised communities (Gonçalves Leite and de Araújo Castro, 2021; Hussein and Nye, 2024).

As of 2024, Colombia had received in excess of 2.8 million Venezuelan migrants—the highest number in the region (R4V, n.d.). The scale of the phenomenon has tested the country's ability to integrate migrants and ensure access to essential services such as food, shelter, and medical care; such challenges have been especially pronounced in border regions (Doocy et al., 2019). The situation has had negative effects on native workers (Caruso, Gomez Canon, and Mueller, 2021), but it has also presented opportunities for growth (Rossiasco and de Narváez, 2023).

The Colombian government has had to confront these challenges amid growing public opposition and increasing rejection of migrants (Invamer, 2021; Hussein and Nye, 2024). To address the situation, the country progressively expanded protections and implemented various regulatory measures for Venezuelan migrants (Besserer Rayas, Finn, and Freier, 2024). The most significant step came in 2021 with the introduction of the Temporary Protection Statute for Venezuelan Migrants (in Spanish: *Estatuto Temporal de Protección para Migrantes Venezolanos*, ETPV), a 10‐year regularisation mechanism that allows Venezuelan migrants access to work, healthcare, and education, and grants them a path to residency. This response has been widely praised, positioning Colombia as a regional leader in managing international displacement (Freier and Gómez García, 2022; Fernández‐Rodríguez and Célleri, 2024). By late 2024, more than two million Venezuelans had been registered by the biometric system and had received stay permits under the ETPV (Migración Colombia, 2026).

This approach has delivered tangible benefits, including increased formalisation and access to services, as well as producing significant migrant contributions to the economy. In 2022, Venezuelan migrants paid USD 529.15 million in taxes, exceeding the USD 494.2 million spent by the government on their healthcare and education (Equilibrium Social Development Consulting, 2024). Regularisation has not only improved migrants' well‐being—enhancing income, consumption, and health outcomes—but has also helped to formalise labour and reduce pressures on public services (Ibáñez et al., 2025). In contrast, border closures implemented in other South American countries, such as in Chile, Ecuador, and Peru, have proven ineffective, as they have exacerbated irregularity (Hammoud‐Gallego, 2024).

Colombia's relative success is even more remarkable considering its recent history. The country endured more than five decades of internal armed conflict involving guerrilla groups, paramilitaries, and drug cartels (Ríos, 2017). This violence led to the internal displacement of more than 7.7 million people and deeply fragmented state authority, especially in rural and border areas (Ríos, 2017; UNHCR, 2018). And yet, it was in this context of protracted crisis and limited institutional reach that Colombia developed core capacities for managing international displacement: management of international aid; inter‐agency coordination; national registration systems; and adaptation of external models—drawing lessons from countries such as Germany, Peru, Turkey, and the United States. Furthermore, it understood the complex nature of the border, including its length, porosity, and the presence of binational armed groups and ethnic communities. These institutional legacies now underpin its response to Venezuelan displacement.

While existing academic research on large‐scale displacement policy has emphasised political determinants—such as foreign pressure, public opinion, and state interests (Zolberg, Suhrke, and Aguayo, 1989; Milner, 2009; Said and Jara, 2022)—less attention has been paid to the institutional dimensions that enable policy implementation. Some studies examine legal frameworks, administrative structures, and bureaucratic competences (Jacobsen, 1996; Blair, Grossman, and Weinstein, 2022; Fernández‐Rodríguez and Célleri, 2024), helping to explain why policies take either liberal or restrictive forms; however, they offer limited insight into the state's ability to adopt effective policies.

Drawing on 40 semi‐structured interviews and a review of literature on internal displacement policies, this research argues that the reason Colombia was able to implement an ambitious and humanitarian policy lies in its institutional legacies from internal displacement. These legacies have facilitated what I term ‘capacity transfer across crises’. Unlike previous studies of international displacement policy (Basok, 1990; Abdelaaty, 2021), I suggest that the Colombian state's ability to navigate the current crisis is thus not solely a matter of political will but also reflects its long‐standing institutional resilience.

In addition to its empirical contribution regarding the factors behind Colombia's response to Venezuelan displacement, this article also makes three key theoretical contributions and one policymaking contribution. First, it advances the study of large‐scale displacement policy determinants by emphasising the role of institutional capacities. Second, it adds to the literature on crisis management by introducing the concept of **capacity transfer across crises and by discussing the application of the ‘vulnerability paradox’ to this case**. In doing so, it enriches and applies crisis management literature, typically focused on Western contexts and shorter‐term types of crises, to international displacement scenarios in Latin America. Third, it contests the prevailing assumption that Latin American institutions are weak (Brinks, Levitsky, and Murillo, 2019), positing that countries perceived as vulnerable owing to ongoing crises can exhibit robust capacities to manage subsequent challenges. Lastly, this work offers important insights for policymaking by highlighting the key governance capacities that contribute to an effective response.

The article continues by introducing the key concepts that underpin the analysis, followed by the application of this conceptual framework to the Colombian armed conflict and Venezuelan displacement, framing both as crises and examining responses as forms of crisis management. The next section outlines the methodology used in the study. This is followed by the empirical analysis, which examines the specific institutional capacities and state responses mobilised by Colombia in the aftermath of Venezuelan displacement. The paper then discusses the findings in relation to existing literature and concludes by summarising the main insights and offering suggestions for future research and policy.

## 2 THEORETICAL TERMS

*Crises* are conceived as critical junctures where a state perceives an urgent threat to its core structures and fundamental values, characterised by significant uncertainty and requiring far‐reaching responses (Boin et al., 2016). According to this framework, they encompass three key components: perceived threat to essential values; the urgency to act; and inherent uncertainty. The large‐scale arrival of migrants fleeing persecution or humanitarian crises—spanning both internal and international displacement scenarios—often meets these criteria, as it poses immediate threats to the well‐being of the displaced, challenges the host society's capacity to absorb them, and creates pressures on public services, labour markets, social cohesion, and food security (Ibáñez and Velásquez, 2008; Ianchovichina and Ivanic, 2014; Dadush and Niebuhr, 2016; George and Adelaja, 2022). Additionally, the unpredictability surrounding the scale, duration, and impact of such displacement entails an inherent uncertainty for host societies and migrants themselves, necessitating swift and coordinated responses across sectors.

Unlike sudden crises, such as terrorist attacks, flash floods, landslides, volcanic eruptions, or earthquakes, large‐scale displacement is often a long‐term phenomenon. It can last for years or even decades, until the situation in the migrants' home region stabilises, thus constituting a protracted crisis (Mencütek, 2019). This prolonged nature changes the urgency, as the host state is expected to prioritise these issues within a sustained time frame, with an ongoing need for adaptation as migration patterns evolve.

Understanding international large‐scale displacement here as a crisis is not about framing it as an inherently negative phenomenon that demands or justifies restrictive measures, as prior research suggests (see, for example, Carastathis, Spathopoulou, and Tsilimpounidi, 2018; Almustafa, 2022); rather, it is about recognising the critical need for robust management. While international displacement can generate positive externalities, including economic contributions and cultural enrichment (Ianchovichina and Ivanic, 2014; Dadush and Niebuhr, 2016; Khatua and Sarma, 2019; Rossiasco and de Narváez, 2023), these outcomes are not automatic and frequently depend on the host country's institutional capacity.

This perspective differs from the approaches of migration studies that treat crisis mainly as a socio‐political construct (Margheritis, 2025a), by emphasising that displacement also constitutes a material and institutional challenge that demands sustained, cross‐sectoral coordination. Nevertheless, it aligns with these constructivist interpretations in recognising that the use of the term ‘crisis’ can be analytically valuable: it captures the long‐term and uncertain nature of displacement, helps to convey its complexity, and may open a window of opportunity for innovative governance, coordination, and external support (Guner, 2025; Margheritis, 2025b; Mencütek and Barthoma, 2025). Rather than idealising displacement or reducing it to a burden, this perspective encourages realistic responses that balance humanitarian needs, mitigate negative externalities within receiving countries, and support the long‐term welfare of both native and migrant communities.

*Crisis management* encompasses formal, structural, and procedural features of governmental administration and informal elements, underlining how these aspects function in practice (Boin et al., 2016). Since crises often strike existing institutions unexpectedly, they require rapid and effective responses derived from pre‐existing capacities. I identify these capacities as ***institutional legacies***; illustrating how institutions and practices developed in response to historical events continue to shape policy responses. Once established, institutions cultivate procedures, organisational norms, and capacities that become embedded within state structures, and persist despite changes in political and social environments. This research emphasises institutional legacies as manifestations of state capacity, rather than as mere structural constraints. Thus, it shifts away from deterministic interpretations of path dependency (Mahoney, 2000; Collier and Collier, 2002), viewing institutional legacies as resources stored in the state's memory that can be activated and adapted to meet new challenges.

In fact, a state that has undergone a crisis may demonstrate greater resilience in adapting to new crises than a state that has never faced such challenges. This observation aligns with the **vulnerability paradox**, which, in the context of states, posits that those that are more vulnerable can exhibit greater resilience to deal with crises than their supposedly less vulnerable counterparts (Campbell and Hall, 2017). However, diverging from Campbell and Hall's (2017) analysis, which links vulnerability to small size, vulnerability in this study is connected to having undergone previous crises. This occurs because some capacities are abstract and applicable to various crises, referred to here as ***generic* capacities, such as the capacity to collect information**; others, though, are *specific* to the nature of crises, such as the development of registration systems. In this regard, the state must demonstrate ***capacity transfer across crises***, positing that institutional capacities developed to manage one type of crisis can be repurposed to address a different kind.

Colombia's response to Venezuelan displacement demonstrates how the state's capacities originally intended to manage internal displacement and the armed conflict were successfully adapted to manage a humanitarian, international displacement crisis. Thus, it can be seen as a form of capacity transfer across crises and a direct result of its institutional legacies derived from the armed conflict.

## 3 BACKGROUND

### 3.1 Armed conflict and internal displacement

The Colombian armed conflict, one of the longest‐running in Latin America, has its roots in political, economic, and social tensions from the mid‐twentieth century. Although political violence dates back to *La Violencia* (1948–58), the conflict escalated in the 1960s with the formation of guerrilla groups like the Revolutionary Armed Forces of Colombia (FARC) in 1964, the National Liberation Army (ELN) in 1965, the Popular Liberation Army (EPL) in 1967, the 19 April Movement (M‐19) in 1970, and the Quintín Lame Armed Movement (MAQL) in 1984.^1^ These groups pursued goals ranging from land redistribution to indigenous rights and democratic reforms (Ríos, 2017).

In the 1980s and 1990s, the rise of drug cartels, especially the Medellín and Cali Cartels, turned Colombia into a key hub of the global drug trade. This illicit industry fuelled the conflict as guerrillas and newly formed paramilitary groups like the United Self‐Defense Forces of Colombia used drug revenues to fund their operations, leading to high levels of violence, massacres, forced displacement of civilians, and heightened human vulnerability (Ríos, 2017).

Throughout the 2000s, the Colombian government launched the ‘Democratic Security Policy’, with the US under ‘Plan Colombia’. Despite some military successes, this strategy was marred by serious human rights violations, notably the *falsos positivos* scandal, in which more than 6,400 civilians were killed and falsely presented as combatants (Comisión de la Verdad, 2022). A major shift occurred with the 2016 peace agreement with FARC, which included mechanisms for reintegration, rural development, and transitional justice. Yet not all factions demobilised; dissident groups such as Segunda Marquetalia and the Gulf Clan remain active.

The armed conflict represents a humanitarian crisis that aligns with the framework set out by Boin et al. (2016), characterised by elements of threat, urgency, and uncertainty. It had devastating consequences: more than 260,000 deaths, the loss of territorial control, a legacy of social trauma, and millions of displaced people (Comisión de la Verdad, 2022). The number of internally displaced persons in Colombia is among the highest in the world (UNHCR, 2018).

The scale and complexity of the armed conflict thus threatened core societal values, including the protection of fundamental rights and the stability of state institutions. Addressing this crisis required sustained yet urgent action to uphold these values and mitigate the profound humanitarian and social impacts. The inherent uncertainty surrounding the scale, duration, and resolution of the crisis made it difficult for authorities and host communities to plan and allocate resources effectively. Despite the peace process, Colombia still faces ongoing challenges, including the reintegration of ex‐combatants, the dismantling of remaining paramilitary and criminal networks, the limited political will for implementation (particularly under the administration of 2018–22), and the urgent needs of displaced communities.

### 3.2 Venezuelan displacement and Colombia's response

The Venezuelan displacement crisis began in 2015 with the deportation of 1,500 Colombians from Venezuela, escalating as tens of thousands of Colombian returnees fled persecution (Fernández‐Rodríguez and Célleri, 2024). This early wave included many Colombian returnees and their descendants, reflecting historical migration during Venezuela's oil boom of the 1970s. By 2017, hyperinflation, basic needs shortages, insecurity, and the curtailment of freedoms by Nicolás Maduro's regime (Gouveia, 2022) led to an influx of up to 37,000 people entering Colombia daily (Mojica et al., 2020). By the end of 2018, 1 million migrants had arrived, a number that grew to more than 1.7 million by 2021 (R4V, n.d.).

Like Colombia's internal displacement, this phenomenon constituted a critical juncture, characterised by a perceived threat to societal core values, urgency, and uncertainty, aligning with Boin et al.'s (2016) crisis framework. Perceived threats emerged from both migrant vulnerabilities and host community concerns. Migrants faced extreme vulnerability—food insecurity, limited healthcare, informal labour, and xenophobia (Doocy et al., 2019; Agudelo‐Suárez et al., 2022)—whereas host societies voiced concerns over pressure on public services, labour market competition, and security (Oxfam International, 2019). While some of these concerns, particularly those related to healthcare and localised labour impacts, were empirically grounded (Caruso, Gomez Canon, and Mueller, 2021; Peñaloza‐Pacheco, 2022), others, like crime, were not supported by evidence (Bahar and Dooley, 2021; Knight and Tribin, 2023).

Even when unfounded, perceived threats generated political urgency, highlighting the need for state‐led responses that balanced humanitarian commitments with socio‐economic concerns. At the same time, policymakers recognised the potential long‐term benefits of integration, including labour market expansion and economic growth (Mutis O. et al., 2021; Rossiasco and de Narváez, 2023).

The uncertainty regarding the duration of the crisis was also notable. As ongoing instability in Venezuela made it unclear how long the displacement would last, the Colombian government's response evolved over time. Initially, the focus was on emergency measures at the border, such as the introduction of the Border Transit Card in 2016, which allowed Venezuelans to enter Colombia without a passport and remain for seven days. In 2017, the government introduced the Special Permit to Remain (in Spanish: *Permiso Especial de Permanencia*, PEP) to regularise the status of Venezuelans in the country, offering a temporary two‐year permit to stay, work, and access public services. The PEP had limitations, however, such as requiring legal entry through official crossings, which many Venezuelans had not used, and its expiration after two years without providing a clear path to permanent residency.

In response to these issues, the government extended the PEP in multiple phases, and in 2018, it introduced an administrative registration system for irregular migrants, including those who had entered illegally or without passports. This programme enhanced migrants' well‐being while reducing costs to the state (Ibáñez et al., 2022). Many Venezuelans remained irregular, though: by June 2020, only 44 per cent were regularised (Presidency of the Republic of Colombia, 2020).

In February 2021, the Colombian government announced the adoption of the ETPV (via Decree 216/2021). The programme included a biometric registration system and a stay permit. This measure was distinct from previous ones owing to its more flexible requirements, allowing migrants who had entered irregularly up to January 2021 to qualify, and extending eligibility to those arriving until May 2022. Furthermore, it introduced the possibility of applying for permanent visas after a 10‐year period.

The adoption of the ETPV was driven by a pragmatic cost–benefit analysis, while safeguarding Colombia's humanitarian values. Policymakers acknowledged the inevitability of migration and its long‐term nature, due to the ongoing crisis in Venezuela, and were aware that regularisation was the best approach to integrate migrants, in line with existing studies (Bahar, Dooley, and Huang, 2018; Ibáñez et al., 2022, 2025). The ETPV aimed to provide migrants with access to rights, improve their conditions, and alleviate the burden on public services, especially healthcare. It also aimed to facilitate labour market participation, boost productivity, reduce informal work, prevent deteriorating employment conditions, and promote economic growth. Additionally, identification and regularisation sought to improve security by enabling better identification and addressing concerns about impunity and human trafficking (Fernández‐Rodríguez, 2024).

## 4 DATA AND METHODS

Colombia was selected as a deviant case owing to its notably liberal response to large‐scale displacement, despite having among the highest number of displaced persons globally (UNHCR, 2024). This contrasts with prevailing global trends, where high inflows typically lead to more restrictive or securitised responses, often resulting in ineffective policies (Mencütek, 2019; Said and Jara, 2022; Hammoud‐Gallego, 2024). Deviant cases—those that defy general theoretical expectations—are especially useful for uncovering alternative causal mechanisms (Seawright and Gerring, 2008). This research uses the anomaly of Colombia to explore whether institutional legacies help to explain this exceptional outcome.

This analysis employs a **process‐tracing approach** to investigate whether and how Colombia's institutional legacies—particularly those developed in response to internal displacement—contributed to the effectiveness of its policy response to Venezuelan displacement. Process tracing enables a within‐case analysis of causal mechanisms, focusing on the sequential and interactive steps that connect institutional antecedents to policy outcomes (George and Bennett, 2005; Beach and Pedersen, 2019).

The analysis identifies key capacities leveraged during ETPV policymaking and traces their origins in past legacies, established during Colombia's response to the armed conflict. It does so by using interview data and secondary sources to assess the plausibility and coherence of the proposed causal pathway. Table 1 summarises the theoretical assumptions that I test with my empirical data by employing process‐tracing.

**TABLE 1:** Causal mechanism linking institutional legacies with effective policy responses to international displacement.

Cause (X)	Part 1	Part 2	Outcome (Y)
Institutional legacies from prior crises.	The state develops institutional capacities during crises.	The state activates and adapts these capacities in response to a new crisis.	Effective policy response to large‐scale displacement.

**Source:** author.

**FIGURE 1:**
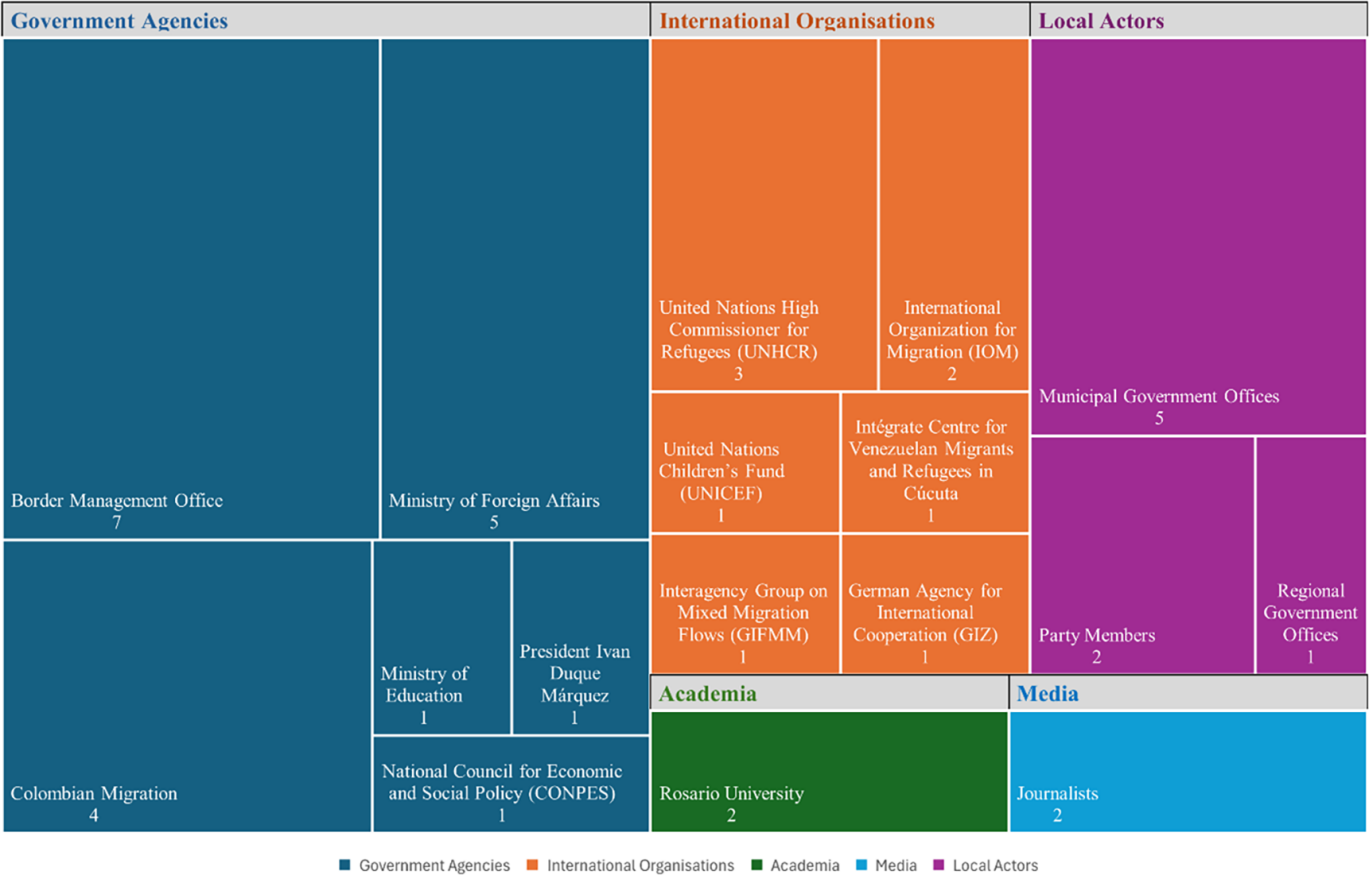
Overview of interviewees. **Source:** author.

Specifically, I conducted 40 semi‐structured interviews with policymakers, including politicians and government officials, and with experts on internal and international displacement, including staff of international organisations, academics, and former government officials, which took place during fieldwork in Colombia between 2022 and 2023. Given the nature of this research, I selected interviewees through a **purposive sampling strategy**, targeting individuals with privileged access to decision‐making processes and knowledge of institutional legacies. I also employed the snowball technique to expand access to high‐ranking politicians and senior administrative officials. Figure 1 summarises the institutions to which the interviewees belong.

The first interviews conducted with policymakers—including members of Colombian Migration, the Border Management Office (BMO; in Spanish: *Gerencia de Fronteras*), the Ministry of Foreign Affairs, and the Presidency—focused on the features of policymaking for the ETPV. I concentrate on this as it highlights an increasing emphasis on the integration of migrants, driven by the perception that many will likely remain in the host country, with long‐term positive effects for both Venezuelan arrivals and the host society (Freier and Gómez García, 2022).

These interviews aimed to identify the capacities that Colombia leveraged in managing the situation, and employed a semi‐structured format, which combined open‐ended questions with probing follow‐ups (Harvey, 2011; Adeoye‐Olatunde and Olenik, 2021). This format permitted exploration of fixed questions on the role of institutional capacities in effective management and provided flexibility for policymakers to reflect on broader aspects of the response. However, policymakers did not always acknowledge how past experiences influenced their decisions and, at times, they denied such connections, reflecting the legal and operational distinctions between internal and cross‐border displacement. This made it particularly challenging to attribute directly specific responses to institutional memory or capacity.

To investigate further potential influences of capacity transfer, I conducted a review of literature on the management of internal displacement, as well as holding additional interviews with former civil servants and representatives of international organisations involved in the response, such as the United Nations High Commissioner for Refugees (UNHCR) and the International Organization for Migration (IOM), responsible for delivering the response to the Venezuelan displacement crisis.

I analysed the empirical material using thematic analysis based on a codebook, which combines a coding frame with early theme development (Braun and Clarke, 2022). Guided by the logic of process tracing, I tracked specific institutional capacities mobilised in ETPV policymaking back to earlier institutional legacies. Rather than applying a coding scheme based on prior theory, I generated the codes from patterns emerging in the empirical data. After familiarising myself with the data, I identified four primary capacities that served as the main categories or codes: ‘understanding of the nature of the border’; ‘inter‐agency coordination’; ‘organisation and management of international agencies’; and ‘population registration’. During the later stages of recoding and refinement, I identified an additional capacity: ‘integration of external models’. To capture the nuances within these categories, I developed subcodes to highlight specific elements. For instance, under the broader code of ‘coordination with international agencies’, I included subcodes such as ‘the role of the BMO’.^2^ These patterns were identified by interpreting recurring ideas across interviews—for example, references to the porous and informal border were classified as ‘understanding of the nature of the border’. Table 2 provides an excerpt from the codebook.

**TABLE 2:** Codebook extract.

Code	Definition	Example
Understanding of the nature of the border	Refers to the knowledge held by migration authorities about border management.	‘Due to the internal conflict, there has been demand from neighbouring countries for security at its borders … particularly security problems. This was especially true for the Cúcuta border with Venezuela, the northern border, which is probably the most dynamic of our borders’.
Inter‐agency coordination	Captures the coordination systems (such as the BMO) relevant for policymaking. Includes state coordination with civil society and international agencies, as well as among different government sectors.	‘What we agreed upon with the government was to have coordination among the international community, civil society, donors, and the government to improve the response to the existing humanitarian crisis’.
Organisation and management of international agencies	Focuses on the authorities’ capacity to manage and coordinate international aid effectively.	‘I believe that a part of Colombia's history is that Colombia is the second country in the world to host UN [United Nations] agencies … international cooperation was very important.’
Population registration	Reflects how identification systems are prioritised in migration management.	‘One of the important aspects of the ETPV is that it provides for a registration system for migrants … regularisation protects both Venezuelan criminals and those who are victims’.
Integration of external models	Relates to the use of international examples during policymaking.	‘We looked for international tools and previous experiences and found only two before ours: the American TPS [Temporary Protected Status] and the Turkish TPS [Temporary Protection Status] … [these] developed into our own study’.

**Source:** author.

**FIGURE 2:**
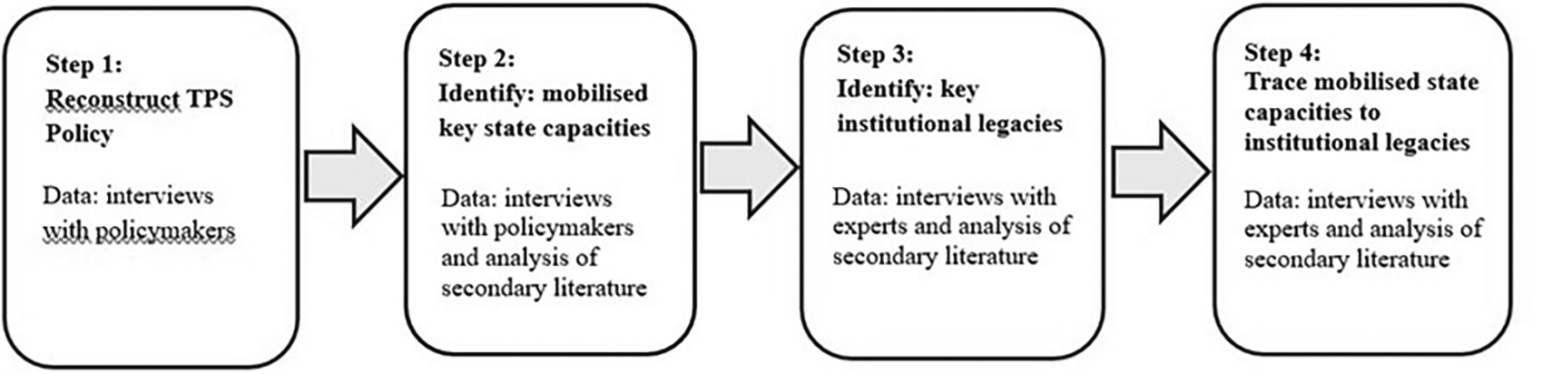
Data analysis steps. **Source:** author.

Figure 2 summarises the methodological steps that I undertook to analyse the data.

The following section presents the results of the empirical analysis. In line with ethical considerations, all interviews are anonymised using a coding system. Each empirical claim—whether based on a direct quotation or an interpretive insight—is followed by a code beginning with the letter ‘I', indicating an interview source. The second part of the code reflects the type of interviewee, using the following categories: GOF (National Government Officials), IOOF (International Organisation Officers), and LROF (Local and Regional Officials). The complete list of interviewees referenced in the text, along with their respective codes and institutional categories, is provided in Table A1 in the Appendix. The use of these categories ensures that all claims can be traced to specific types of expertise or organisational perspectives, consistent with best practices for qualitative research transparency (Moravcsik, 2014; Kapiszewski and Karcher, 2021).

## 5 RESULTS

### 5.1 Obtaining and managing international aid

Colombia's long‐standing experience in receiving and managing international aid played a key role in shaping its response to Venezuelan displacement. Decades of close interaction with donors—initially linked to the armed conflict and peacebuilding—helped to build institutional trust and strengthen state capacity in aid coordination. These pre‐existing relationships and operational practices were reactivated in the new crisis, not only facilitating rapid mobilisation of support but also informing the policy approach adopted (I‐GOF2, 2022; I‐GOF14, 2022; I‐GOF8, 2023; I‐Duque, 2023).

Colombia hosted a greater number of international aid agencies than most other countries in the region. In 2018, it ranked second globally in the number of United Nations (UN) agencies present (I‐GOF15, 2022; I‐GOF9, 2023). UNHCR opened its first office in the country in 1997. As a government official noted: ‘they did not have to assess whether there was any disagreement with the government and they quickly assigned a person from the UN [Eduardo Stein]’ (I‐GOF15, 2022). This continuity illustrates how institutional legacies—formed in response to earlier crises—enabled Colombia to activate rapidly previously established networks and frameworks in a new crisis context.

Moreover, Colombia has been actively seeking international aid since the 1990s. It launched the *Diplomacy for Peace* initiative, multiplying international support—from USD 100 million to USD 500 million—to alleviate the effects of the armed conflict and foster peacebuilding (García Duque and Casadiego, 2021). As one policy advisor put it, these experiences taught the Colombian state ‘to ask for money’ (I‐LROF1, 2023).

The international aid community exerted significant influence on public policy, as highlighted by García Duque and Casadiego (2021, p. 2382), who describe government intervention as consistently ‘followed by the support, guidance, and supervision of multiple international actors’. The Colombian government was more successful at securing funds from donors for its response than other countries in the region (Freier, Micinski, and Tsourapas, 2021). Under the 2019 Refugee and Migrant Response Plan, it obtained 51 per cent of the USD 381 million allocated in total to the region for Venezuela. Furthermore, while Colombia secured 62.1 per cent of its requested funds, the other main recipients received less, with Peru, for example, receiving only 35.9 per cent (R4V, 2020). This reflects Colombia's ability to project crisis governance legitimacy—potentially a manifestation of the ‘vulnerability paradox’, in which past crisis exposure contributes to the development of state capacity.

A key institutional innovation was the creation of the BMO to coordinate the response to Venezuelan displacement, further reflecting Colombia's ability to obtain and manage international aid. Located at the presidential level, the BMO was entirely funded and staffed through international aid sources, demonstrating both Colombia's ability to mobilise external support and its embeddedness in transnational governance networks. International aid not only contributed to financial and logistical resources but also played a strategic role in shaping the policy approach—providing the government with evidence of the benefits of regularisation and integration. As a former government official explained, the contribution of international actors during the ETPV policymaking process was ‘more a source of knowledge than of funding’ (I‐GOF9, 2023).

Another official traced the ability of the Colombian government to use international aid to its previous experience of managing internal forced displacement:

*The international aid community had a historical presence, so here in the Department [of Norte de Santander] we had assistance from the Norwegian [Refugee] Council, [which is] very good in educational matters. We have been working with displaced people for maybe 20 or 25 years, and we had developed pedagogical models, addressing crucial topics within the conflict. The Norwegian [Refugee] Council was already here when the migration crisis started, and we wanted it to be there because of its expertise. Clearly, Colombia had a stronger pull compared to other countries because it already had a deep understanding and a long‐standing relationship with international aid* (I‐GOF11, 2022).

This reflects what I define as a form of ‘generic’ institutional capacity—abstract skills in international negotiation, project implementation, and donor coordination—that are transferable across different types of crises. In Colombia's case, the shift from an armed conflict to an international displacement crisis did not require building new systems from scratch, but rather repurposing existing ones, illustrating how institutional legacies can become institutionalised and reactivated, and how states with a history of vulnerability can evolve into effective crisis managers.

### 5.2 Coordinating international, national, and local actors

In response to crises that strained security, services, and economic stability, successive administrations developed centralised structures to align national, departmental, and municipal responses. As part of the National System for Comprehensive Assistance and Reparation for Victims, established by Law 387/1997, the government created the National Council for Comprehensive Care of Displaced Populations, which brought together various ministries to develop policy and support local efforts. In parallel, the Social Solidarity Network (RSS, the Spanish acronym) was created by Law 368/1997 to coordinate multi‐level responses to internal displacement. These efforts were later consolidated under the Presidential Agency for Social Action and International Cooperation, by merging the RSS and the Colombian Agency for International Cooperation (Decree 2467/2005). This reform expanded the agency's mandate to broader conflict‐related issues and marked a shift towards integrating international support into domestic crisis governance. Together, these developments reflect an evolving state capacity to coordinate national and international actors within a centralised framework.

The BMO mirrors previous presidential‐level coordination and decision‐making efforts (I‐LROF1, 2023). Like the internal displacement crisis, the influx of forcibly displaced Venezuelans has impacted multiple state sectors, including healthcare, education, and security. The establishment of a Management Officer is a recurring, although informal, practice in Colombia to address economic crises or environmental disasters (I‐GOF3, 2022). However, the BMO transcended its traditional crisis management role to assume policymaking responsibilities informally after its formal establishment through Decree 1185/2021 (I‐GOF1, 2022; I‐GOF2, 2022). Composed of technocrats, it operated across sectors and maintained regular coordination with line ministries. The institution played a prominent role in policymaking, with its proximity to the presidency empowering BMO bureaucrats in the process (Fernández‐Rodríguez and Célleri, 2024). One government official noted:

*People from the Ministry of Foreign Affairs do not know how to manage resources; they can communicate with donors but need technical staff who understand the issues and can provide strong leadership. This is what the BMO had. It possessed political capacity and expertise, enabling it to navigate complex situations. The Ministry of Foreign Affairs lacks the mandate to mobilise other ministries, focusing primarily on international relations, while many migration issues intersect with domestic policy concerning services and economic integration* (I‐GOF4, 2022).

Colombian authorities learned from the armed conflict that engaging local actors in policymaking and implementation is crucial to preventing the erosion of state authority by armed groups. As a result, territorial committees, led by mayors and governors, emerged as key institutions in addressing the internal displacement crisis. Operating through local response plans, these committees identified challenges, developed intervention strategies, and provided direct assistance to forcibly displaced individuals at the regional and local levels (Ibáñez and Velásquez, 2008).

Drawing on this model, the Colombian government established migratory boards in 2018 in regions and municipalities with high numbers of forcibly displaced Venezuelans (I‐IOOF1, 2023; I‐LROF1, 2023). Similar to the territorial committees, these boards aimed to coordinate efforts among international, national, and local authorities while assessing local challenges to inform national responses. These boards mirrored the earlier structures in form and function, coordinating among international, national, and local actors, and informing national‐level decisions with bottom‐up assessments. By 2020, there were 19 regional, 4 sub‐regional, and 5 municipal migratory boards operating across the country (Presidency of the Republic of Colombia, 2020); interviewees described them as critical to a harmonised response (I‐GOF1, 2022; I‐GOF11, 2022).

The international aid community also adapted its coordination mechanisms by building on structures first developed during Colombia's internal displacement crises. The **Protection Cluster**, led by UNHCR since 2006 in partnership with the Norwegian and Danish Refugee Councils, served as a foundational model. It later inspired the creation of the **Interagency Group on Mixed Migration Flows (GIFMM, the Spanish acronym)** in 2016, which aimed to coordinate humanitarian responses to Venezuelan displacement. Interview data highlight that coordination through the BMO, migratory boards, and GIFMM enabled a more targeted policy design. These mechanisms helped to produce sector‐specific assessments and more accurate diagnoses, resulting in better tailored interventions. According to interviewees, communication between international, national, and local actors, through the establishment of the BMO, migratory boards, and the GIFMM, was crucial for providing accurate determinations on the impact on various sectors and appraising the advantages and disadvantages of different policy models (I‐GOF1, 2022; I‐GOF11, 2022; I‐GOF15, 2022; I‐LROF2, 2022; I‐GOF7, 2023; I‐Duque, 2023). One government official taking part in these processes emphasised the importance of this institutionalisation in Colombia's response:

*The BMO played a coordinating role, but the government created several bodies—not just the BMO, but also the migratory boards. National‐level problems couldn't be resolved in isolation. Within each institution, there was a designated person at both the national and local levels, and international aid was coordinated through the GIFMM. This institutional design facilitated Colombia's progress in responding to these challenges. I genuinely believe that these responses were not merely political decisions: they were the result of organised action demonstrating effective coordination within the government* (I‐GOF1, 2022).

This coordination model reflects a clear case of capacity transfer across crises: institutional mechanisms originally designed to manage internal displacement and the armed conflict were adapted to a new displacement context, reinforcing the state's ability to manage complex crises through multi‐level coordination and the centralisation of decision‐making.

### 5.3 Establishing registration systems

Another important lesson taken from internal forced displacement is the importance of identifying the affected population—an element that stands out in Colombia's response to Venezuelan displacement. In 2000, the government established a Unified Registration System for Displaced People, which documents and identifies victims of forced displacement. This framework was later expanded with the creation of the Unified Registration System for Victims (RUV, the Spanish acronym), which includes information on both internally forcibly displaced people and other victims of the armed conflict.

The experience gained from addressing internal forced displacement highlighted the importance of effectively identifying forcibly displaced Venezuelans through an accurate registration system. In 2018, Colombian authorities implemented the Administrative Registry of Venezuelan Migrants (RAMV, the Spanish acronym; via Decree 542/2018), a socio‐demographic registry designed to pinpoint the needs of Venezuelans lacking regular status. Similar to the RUV, these efforts aimed to inform international displacement policy (Presidency of the Republic of Colombia, 2020). However, the registration process lasted for only two months and lacked biometric data. As forcibly displaced Venezuelans continued to arrive, the information gathered through the RAMV became outdated. The ETPV aimed to address these issues, with its biometric registration seen as necessary for guiding further responses to the crisis. A policymaker connected Colombia's experience of forcibly displaced people to the registration mechanism under the ETPV:

*I believe that our experience with the victims of the conflict, the reintegrated individuals, and those internally displaced showed us the importance of identification, to know, from a budgetary perspective, how much allocation is needed and how many they are. This taught me that we had to identify the population. It was smart to regularise [Venezuelans] because that allowed us to have a profile of the population (I‐GOF8, 2023)*.

Another interviewee considered the registration mechanism for internal forcibly displaced persons to be ‘the most important lesson’ in response to Venezuelan migrants (I‐GOF12, 2023).

This reflects a clear case of capacity transfer across crises—perhaps one of the most *specific*—where tools developed for managing internal displacement during conflict were adapted to a new humanitarian crisis, structuring a more durable and data‐driven approach to cross‐border migration.

### 5.4 Adaptation of external models

Another key institutional legacy mobilised during the ETPV policymaking process was Colombia's established capacity to draw on foreign experiences of responding to crisis situations. It relied on the international community during its peacebuilding endeavours to reach agreements with armed groups. Under its Diplomacy for Peace initiative, Colombia began actively seeking international support for negotiations with armed groups. This was followed by periods of more limited engagement, before international involvement was again emphasised to enhance the credibility and effectiveness of peacebuilding efforts (Barreto, 2014).

Peacebuilding processes were frequently conducted outside of Colombia, beginning with dialogues with the ELN in Caracas, Venezuela (1991) and Tlaxcala, Mexico (1992), followed by exploratory talks in Mainz, Germany (1998) (Vargas Velásquez, 2014). Negotiations with FARC culminated in formal talks first in Oslo, Norway (2012) and then in Havana, Cuba (2012–16), which ultimately led to the signing of the 2016 peace agreement. Chile and Venezuela served as facilitators, with Cuba and Norway acting as guarantors. In particular, Norway's extensive experience of peacebuilding processes—ranging from Guatemala to Palestine—was regarded as highly valuable. According to Barreto (2014), the Colombian government might have had the Oslo Accords between Israel and Palestine in mind when choosing Norway as the host of the agreements. International organisations such as the European Union and the UN also provided multifaceted support. As both the peace agreement and the beginning of Venezuelan displacement happened concurrently, some of the authorities involved in the Havana negotiations, such as the Chancellor under the administration of President Juan Manuel Santos (2010–18) (I‐GOF10, 2023), were responsible for responding to Venezuelan displacement. This overlap led them to mobilise capacities developed in one process to address the challenges of the other (I‐GOF12, 2023).

The first regularisation mechanism, the PEP, implemented in 2017 via Resolution 50.307/2017, was inspired by a mechanism introduced by the Peruvian government, because of a close collaboration between Colombian Migration and the Peruvian Superintendency of Migration (I‐GOF1, 2022; I‐GOF5, 2023). In May 2017, the Colombian government sent a delegation headed by the Director of Borders to the Turkey–Syria border to examine its response to Syrian displacement. From that experience, Colombian authorities further learned the importance of identifying the Venezuelan population (I‐GOF10, 2023). A former government official also considered lessons from Germany's experience of hosting Syrian refugees, such as the value of distributing refugees across different regions of the country (I‐GOF13, 2023).

Later, the Directorate of Migration and Consular Affairs began to conduct a study on other hosting mechanisms in the US, specifically examining the Temporary Protected Status designation, which allows nationals from countries deemed unsafe by the Department of Homeland Security to work in the country and prevents them from being deported for a limited time (Contreras et al., 2025).

To draft the ETPV, Colombian Migration also sought international experience. Authorities assessed Turkey's model, which granted a significant number of forcibly displaced people from Syria temporary residency permits. A policymaker acknowledged these influences: ‘We searched for tools at the international level, and we only found two: the United States’ TPS [Temporary Protected Status] and the Turkish TPS [Temporary Protection Status]. Those were the only experiences that existed in the world’ (I‐GOF7, 2023).

### 5.5 Understanding the nature of the border

As a result of the armed conflict, Colombian authorities understand the nature of their border with Venezuela as ‘porous’, highlighting in particular the presence of transnational communities such as the Wayú ethnic community and criminal organisations, as well as its 2,219‐kilometre length (Contreras, 2019). This understanding has informed their response to Venezuelan displacement, as they recognise the challenges associated with managing a border characterised by significant movement and interaction.

During the 2000s, the armed conflict in Colombia began shifting towards the border regions—a dynamic often described as ‘peripheralisation’. This transition was driven by state efforts to reassert control over central areas of the country, particularly through security and counterinsurgency strategies. As a result, guerrilla and criminal groups relocated their operations to the Colombia–Venezuela border, with significant activity in regions such as Arauca, César, and Norte de Santander. They found refuge on the Venezuelan side, supported by the Bolivarian National Guard and Army of Venezuela, while the Catatumbo Moist Forests in Norte de Santander became a significant corridor for drug trafficking, further financing these groups. Recognising the evolving security landscape, Colombian authorities acknowledged the need to increase the state's presence in these border areas (Ríos, 2017). A former politician (I‐GOF10) emphasised the critical role of the Venezuela–Colombia border in President Santos' political agenda, stating that: ‘All eyes are looking at the border, as it serves as a prime location for drug trafficking to operate as an exit route’ (I‐GOF10, 2023). As a result, the authorities created institutions around the border, including the Directorate of Borders within the Ministry of Foreign Affairs, which was formally established in 2016 (Resolution 869/2016) (I‐GOF12, 2023).

Faced with Venezuelan displacement, Colombian authorities adopted an adaptive and inclusive approach, drawing on past crisis management **at the border**. In August 2015, the government assigned the response to officials familiar with the Venezuela–Colombia border, including the Director of Borders and the Director of Colombian Migration. Leveraging their knowledge of the region, they developed a border policy aimed at enhancing security and state presence. As one interviewee noted: ‘Closing the borders would be a massive logistical and military effort, which would not help protect Venezuelans, but would make the situation of insecurity worse’. While such a move might have satisfied public opinion, he argued, ‘three months later we would have encountered terrible consequences’ (I‐GOF11, 2022). Authorities understood the ‘porous’ nature of the border made closures unrealistic and counterproductive for security (I‐GOF11, 2022; I‐GOF6, 2023; I‐ IOOF2, 2023). Such closures would only push people towards irregular crossings (*trochas*), enriching guerrilla, paramilitary, and criminal groups such as the ELN and the Gulf Clan (which calls itself the Gaitanist Self‐Defense Forces of Colombia).

**FIGURE 3:**
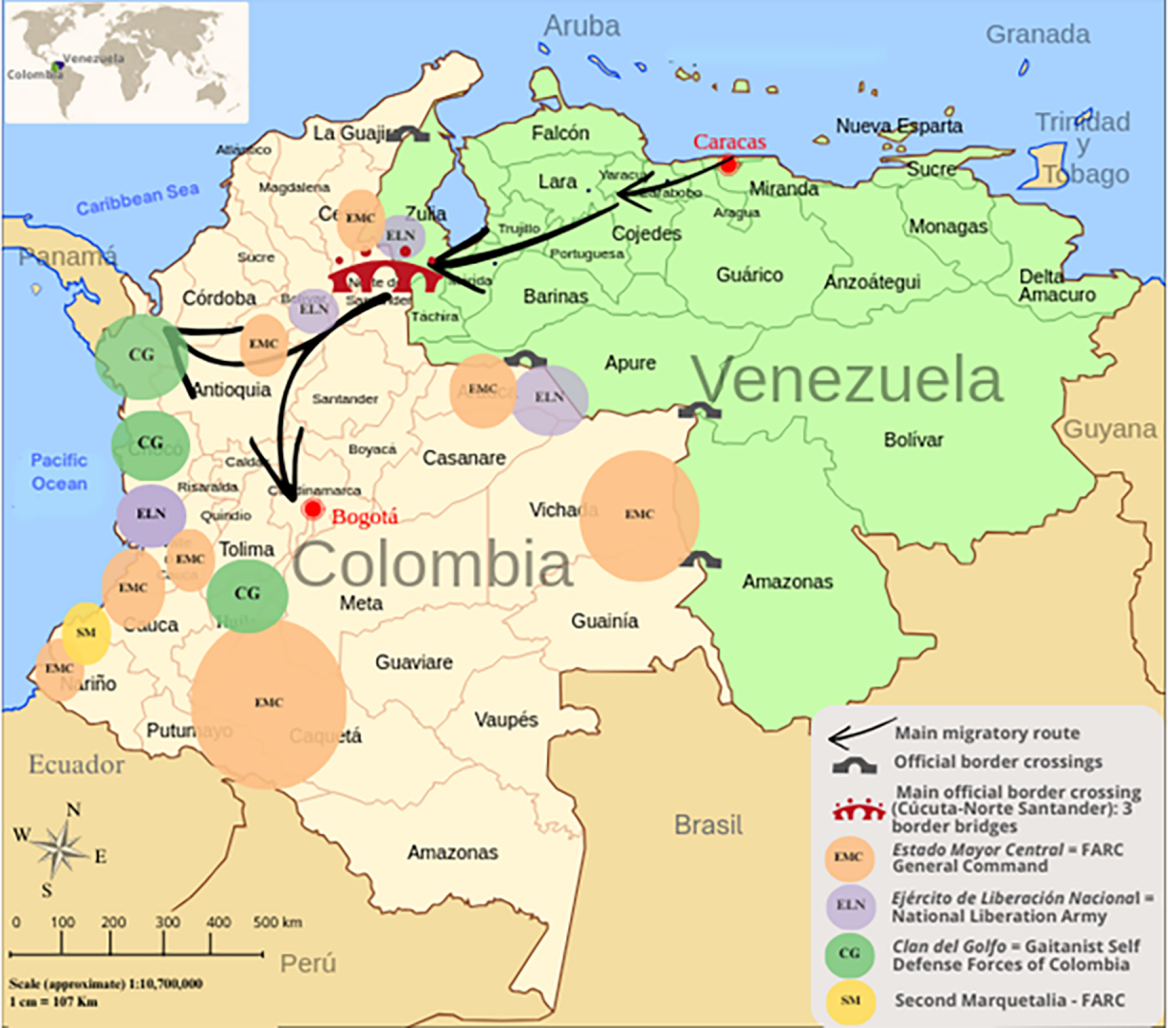
Main migration routes from Venezuela and armed group presence in Colombia **Source: ‘**Colombia Venezuela Map’ by Rαge, with derivative work by Shadowxfox (https://commons.wikimedia.org/wiki/File:Colombia_Venezuela_map.svg). Elements of my own creation have been added, and for the presence of armed groups I have relied on the graphic by Fundación Ideas para la Paz (2024). Licence: CC‐BY‐SA‐3.0 (https://commons.wikimedia.org/wiki/Category:CC‐BY‐SA‐3.0).

Figure 3 shows the main migration routes from Venezuela and the presence of armed groups in Colombia, reflecting the complexity of border dynamics.

**This case illustrates another form of capacity transfer across crises**: the expertise on the border developed in response to the armed conflict was repurposed to address a new, cross‐border humanitarian crisis. It clearly exemplifies the **vulnerability paradox**: repeated exposure to crisis conditions, far from weakening the state, generates embedded knowledge and resilience that can be mobilised in future emergencies.

## 6 DISCUSSION

Migration literature has long cautioned against framing international displacement as a ‘crisis’ (see, for example, Carastathis, Spathopoulou, and Tsilimpounidi, 2018; Almustafa, 2022), warning that this can stigmatise migrants and obscure the structural causes of their movement as well as states' inability to integrate them effectively. While these concerns are valid, this research argues that when used carefully, the crisis lens remains analytically useful, particularly for understanding the actual challenges that large‐scale displacement presents to the receiving country and how the state responds under pressure. In Colombia's case, the government's acknowledgement of Venezuelan displacement as a challenge to institutional and social stability prompted exceptional policy responses, including institutional adaptation and inclusive policymaking—a dynamic comparable to Turkey, where crisis framings around the Syrian exodus similarly opened space for policy innovation (Guner, 2025; Mencütek and Barthoma, 2025).

Despite a perception of weakness owing to its history of conflict and internal displacement, Colombia's response to Venezuelan displacement has been remarkably effective. Rather than passively applying existing tools, the government engaged in selective adaptation. Mechanisms developed during internal displacement, such as population registration or inter‐agency coordination (for instance, through the BMO), were reconfigured to fit the distinct challenges of Venezuelan migration. This is a compelling example of the ‘vulnerability paradox’, where a seemingly fragile state demonstrates resilience and efficiency in crisis management. Colombia's experience challenges the notion that only ‘strong’ or ‘stable’ states can effectively handle crises, suggesting instead that a history of adversity can cultivate precisely the crisis management skills and adaptability necessary for resilience, and sometimes lead to them even outperforming more stable states that are less experienced in crisis management.

This case also points to the **conditions that might have enabled institutional adaptation**. First, **temporal proximity** between crises allowed for the persistence of bureaucratic memory and continuity of personnel—several officials managing Venezuelan displacement had previously worked on internal displacement. Second, **political will**, particularly between 2018 and 2022, played a crucial role in supporting technical responses over securitised ones. At the same time, institutional learning was **not automatic**: several capacities were adapted informally and only later institutionalised (such as the BMO), and some actors denied any link with past experiences, suggesting that learning was sometimes tacit rather than explicit.

## 7 CONCLUSION

Colombia's response to Venezuelan displacement demonstrates how institutional legacies from previous crises—particularly experience of internal forced displacement—can be effectively adapted to manage new and complex forms of migration. The state mobilised a diverse set of tools: first, its capacity to manage international aid, developed during earlier humanitarian emergencies; second, inter‐agency coordination mechanisms that allowed for a unified institutional response; third, population registration systems originally designed to identify and support internally displaced persons; fourth, the ability to draw on external models and international policy experiences; and fifth, a deep understanding of the country's porous and conflict‐affected border regions.

This case exemplifies the ‘vulnerability paradox’: a state often viewed as fragile responded with notable resilience. Colombia's history of conflict and internal displacement, rather than impeding action, enabled the rapid deployment and adaptation of crisis management capacities. By critically applying the crisis frame, the government was able to activate these institutional resources and develop a relatively effective and humane policy response.

Ultimately, Colombia's experience challenges conventional assumptions about state weakness and resilience. It shows how past adversity can generate adaptive strengths, offering important insights into how states in the Global South can govern large‐scale displacement.

## 8 RECOMMENDATIONS

This research into Colombia's response to international displacement provides crucial information on how states can navigate large‐scale migration through strategic activation of institutional legacies. It also shows, however, that the transfer of crisis management capacities across different contexts is not automatic. In Colombia, key enabling conditions included bureaucratic continuity, which preserved institutional memory, and high‐level political commitment to inclusive approaches. These elements made it possible to adapt tools originally developed for internal displacement and the armed conflict to the new context of cross‐border displacement.

That said, not all countries will share Colombia's institutional history or political environment. In contexts where such legacies are absent or fragmented, efforts to replicate the approach may require investing in capacity building, fostering inter‐agency trust, or creating new frameworks for engagement with international partners. Moreover, Colombia's experience also highlights the importance of flexibility: some institutional arrangements, such as the BMO, began as informal solutions and were only formalised later. Policymakers should thus remain open to adaptive experimentation, particularly in fluid crisis environments.

Finally, while Colombia demonstrates the potential of cumulative crisis experience, it also reveals its limits. Institutional legacies may become outdated or politically contested, and reliance on past models must be balanced with responsiveness to new dynamics. For other countries navigating displacement, the lesson is not to copy Colombia wholesale, but to assess which capacities—technical, organisational, or relational—can be meaningfully adapted to their own institutional realities.

What countries should be studied next? This research raises important questions about the generalisability of the vulnerability paradox and the conditions that enable capacity transfer across crises. Future investigations could compare Colombia's response with that of countries that have not experienced prior large‐scale displacement—such as Costa Rica, which has managed substantial migration from Nicaragua—to assess whether previous crisis exposure fosters greater institutional resilience. Lebanon provides a useful contrast: despite hosting Palestinian refugees for decades, its response to the Syrian refugee crisis has been uneven, making it a valuable case for examining the enabling (or constraining) conditions for institutional learning (BouChabke and Haddad, 2021). Countries with different crisis legacies could also offer revealing insights. For instance, Mexico, which has experienced sustained internal violence, now faces increasing migration from Central America and the Caribbean. Similarly, the Dominican Republic, historically affected by environmental disasters and political instability, must address ongoing migration from neighbouring Haiti. These comparisons would help to clarify whether certain capacities, such as population registration, are more easily transferable across crisis types than broader skills like coordination or inter‐agency planning.

## CONFLICT OF INTEREST STATEMENT

I declare that there is no conflict of interest in relation to publication of this manuscript.

## FUNDING

This research was funded by the WZB Berlin Social Science Center and the SCRIPTS Cluster. The open access publication was funded by the WZB Berlin Social Science Center.

## Data Availability

The data that support the findings of this study are available on request from the corresponding author. The data are not publicly available due to privacy or ethical restrictions.

## Endnotes

^1^
All acronyms based on the names in Spanish.

^2^
The whole codebook is available from the author on request.

## APPENDIX A

### A.1

**TABLE A1:** Interviewees referenced in the text.

Interviewee type	Acronyms used	Place and date of interview
Government Officials	I‐GOF1	Online, 19 December 2022
	I‐GOF2	Bogotá, 21 November 2022
	I‐GOF3	Online, 9 September 2022
	I‐GOF4	Online, 5 December. 2022
	I‐GOF5	Bogotá, 11 January 2023
	I‐GOF6	Bogotá, 21 January 2023
	I‐GOF7	Bogotá, 17 January 2023
	I‐GOF8	Bogotá, 17 January.2023
	I‐GOF9	Bogotá, 9 February 2023
	I‐GOF10	Bogotá, 24 January 2023
	I‐GOF11	Cúcuta, 22 December 2022
	I‐GOF12	Cúcuta, 21 February 2023
	I‐GOF13	Online, 24 January 2023
	I‐GOF14	Online, 28 December 2022
	I‐GOF15	Bogotá, 29 November 2022
International Organisation Officers	I‐IOOF1	Bogotá, 8 February 2023
	I‐IOOF2	Online, 8 February 2023
Local and Regional Officials	I‐LROF1	Bogotá, 20 February 2023
	I‐LROF2	Cúcuta, 8 November 2022

**Source:** author.
